# Grand Challenges in Fungal Genomics and Evolution

**DOI:** 10.3389/ffunb.2020.594855

**Published:** 2020-10-15

**Authors:** Toni Gabaldón

**Affiliations:** ^1^Barcelona Supercomputing Center (BCS-CNS), Barcelona, Spain; ^2^Institute for Research in Biomedicine (IRB Barcelona), The Barcelona Institute of Science and Technology, Barcelona, Spain; ^3^Institució Catalana de Recerca i Estudis Avançats (ICREA), Barcelona, Spain

**Keywords:** genomics, evolution, fungi, yeast, omics

## The Grand and Detailed Views of Fungal Evolution

Sequences are the primary source of information for molecular evolution studies, and now we have nearly unlimited access to them. In recent years, the backbone of the fungal tree of life (fTOL) has been improved, yet some of its lineages are still poorly sampled and our knowledge of the early branching patterns is incomplete (Naranjo-Ortiz and Gabaldón, 2019a). Solving the problem of missing lineages is likely to be only a question of time and perseverance, and we can expect sooner than later that we will have representatives for the major fTOL lineages. However, it is also likely that this alone will not suffice to resolve all the hurdles in the fTOL. Phylogenomic analyses are rife with problems that cannot be solved with just more data (Jeffroy et al., 2006), and resolving particular aspects of the fTOL will certainly remain a challenge for years to come. Related challenges to solving the evolutionary backbone of fungi, is to understand how genomes changed over time, and how these changes impacted the major ecological, and morphological transitions in fungi, including terrestrialization and the origin of multicellularity (Kiss et al., 2019; Naranjo-Ortiz and Gabaldón, 2019b; Gabaldón, 2020). As the number of sequenced genomes increases, we can assess evolutionary relationships at higher levels of resolution. However, as when zooming in into a fractal image, resolution does not necessarily imply solution. For instance, as more genomes become available for a given species, we have the need to consider the concept of pan-genome for fungal organisms (Naranjo-Ortiz and Gabaldón, 2020). In addition, we keep confronting the contentious issue of how to define a species, and the access to more genomes has revealed intricate patterns of complex species relationships, where introgression and hybridization seem not uncommon (Morales and Dujon, 2012; Gabaldón, 2020). Thus, better understanding the processes of genome evolution, adaptation, and speciation, as well as finding a new framework in which to interpret the fungal species concept, are among the immediate challenges ahead of us.

## The Ecology of Fungi

Fungi do not exist in isolation and are an integral part of microbial communities that inhabit all types of niches, including those on or within animals and plants (Frey-Klett et al., 2011; Peay et al., 2016). Genomics, through the analysis of nucleic acids extracted from complex environments—i.e., metagenomics –has revolutionized the way in which microbial ecosystems can be studied, which has many applications including those related to human and animal health, agriculture, environment, and industry. Unfortunately, state of the art methodologies are still mostly focused on the bacterial component, neglecting the ubiquitous but difficult to study fungal component of the microbiome—the so called mycobiome. Fungi are generally present in minor amounts as compared to bacteria, but they have been shown to play pivotal roles in many microbial ecosystems. Yet, their low proportions, the presence of a cell wall, and the lack of appropriate universal markers and reference databases makes it difficult to study them and severely limits our understanding of the microbial ecosystem. Overcoming these challenges will certainly be instrumental to fully exploit the possibilities brought about by metagenomics. The study of the role of fungi in diverse ecosystems, and the analysis of their interactions with other organisms will certainly be a fruitful area of research in the coming years, as it is a highly unexplored frontier.

## Functional Genomics and the Other “OMICS”

Our ability to extract knowledge from sequences depends on how much we can relate it to functional information. In this regard, one of the main challenges facing biology is to assign functions to the ever-growing number of hitherto uncharacterized genes produced by genome sequencing. A growing number of *in silico* approaches allow to provide increasingly informed guesses of what the function of an annotated gene might be (Friedberg, 2006). Besides, there have been many developments in high throughput strategies to screen for gene function and phenotypes in increasingly sophisticated settings, which couple to new genome editing technologies are revolutionizing the experimental characterization of genes (Sharon et al., 2018). In this regard, genomics is increasingly being accompanied with several other large-scale type of data including transcriptomics, proteomics, metabolomics, epigenomics, phenomics, and a long list of other “omics” technologies characterized by their comprehensiveness and highly quantitative nature. As a result, there is an increasing need to integrate all these types of data, and usually this is done by using the genome as a hub, naturally relating all types of information to specific loci. All these developments notwithstanding, there is still a large bias toward a few fungal species models that are not representative of the vast diversity of fungi. Exploring that immensity, or at least a well-thought, representative fraction of it, and do it beyond genome sequences, constitutes a serious challenge that will probably require concerted efforts by many research groups.

## Coping With the Data Flow

Not so long ago, researchers were struggling with the possibility of sequencing a single gene. Now sequencing complete genomes is not the bottleneck of any project anymore and is even used as a starting point. The pace at which new genomes, or raw sequencing data are being deposited in public databases is accelerating in parallel to the increasing throughput capacities of the sequencing machines. The bottleneck now is not producing the data, but rather to analyze it, and later to store it in a way that can easily be found and reused for future projects. Bioinformatics is the inseparable other side of the coin of genomics that we must not forget is under constant need to reinvent itself due to the new types and scales of data. After all, genomics is a moving target, that is also growing as it moves. Although this challenge might seem uninteresting to many who might be tempted to downgraded it as a “simple” technical problem, it is of the upmost importance, as how much we can harness from the wealth of data available largely depends on how well is documented, stored, and inter-connected. Publicly available data repositories, as well as user-oriented web interfaces to obtain and operate with this data, are therefore crucial. The sad challenge that slows us down from achieving this goal is the struggle to get sufficient funding of many user-oriented databases and web-interfaces. Therefore, journals and scientists need to carefully handle the responsibility of making sure all produced data reaches these repositories with a sufficient level of data curation and accessibility. In addition, the software itself must cope with increasing amounts of data of diverse types. Being efficient in the integration of different types of data is a vital a priority. The parallelization and use of large computing infrastructures are thus likely to be more essential, to address some of the research challenges mentioned above.

These four grand challenges are certainly not the only ones ahead of us, but they serve to illustrate the current momentum in the field of fungal genomics and evolution, and the urge to move from sequence data to knowledge, and from piecemeal advances, to unifying perspectives.

## Author Contributions

The author confirms being the sole contributor of this work and has approved it for publication.

## Conflict of Interest

The author declares that the research was conducted in the absence of any commercial or financial relationships that could be construed as a potential conflict of interest.
